# A pilot study of eDNA metabarcoding to estimate plant biodiversity by an alpine glacier core (Adamello glacier, North Italy)

**DOI:** 10.1038/s41598-020-79738-5

**Published:** 2021-01-13

**Authors:** Claudio Varotto, Massimo Pindo, Elena Bertoni, Christian Casarotto, Federica Camin, Matteo Girardi, Valter Maggi, Antonella Cristofori

**Affiliations:** 1 Research and Innovation Centre, Fondazione Edmund Mach, San Michele All’Adige, TN Italy; 2 Uppsala University, Uppsala, Sweden; 3 Geology Department, Science Museum (MUSE), Trento, Italy; 4 Earth and Environmental Sciences, University of Milano Bicocca, Milan, Italy

**Keywords:** Biodiversity, Climate-change ecology, Molecular ecology, Stable isotope analysis, Biodiversity, Climate-change ecology, Ecological genetics, Molecular ecology, Stable isotope analysis, Climate sciences, Plant sciences, Plant ecology, Plant genetics, Plant molecular biology

## Abstract

Current biodiversity loss is a major concern and thus biodiversity assessment of modern ecosystems is compelling and needs to be contextualized on a longer timescale. High Throughput Sequencing (HTS) is progressively becoming a major source of data on biodiversity time series. In this multi proxy study, we tested, for the first time, the potential of HTS to estimate plant biodiversity archived in the surface layers of a temperate alpine glacier, amplifying the *trnL* barcode for vascular plants from eDNA of firn samples. A 573 cm long core was drilled by the Adamello glacier and cut into sections; produced samples were analyzed for physical properties, stable isotope ratio, and plant biodiversity by eDNA metabarcoding and conventional light microscopy analysis. Results highlighted the presence of pollen and plant remains within the distinct layers of snow, firn and ice. While stable isotope ratio showed a scarcely informative pattern, DNA metabarcoding described distinct plant species composition among the different samples, with a broad taxonomic representation of the biodiversity of the catchment area and a high-ranking resolution. New knowledge on climate and plant biodiversity changes of large catchment areas can be obtained by this novel approach, relevant for future estimates of climate change effects.

## Introduction

The current biodiversity loss is a major concern on a planetary level and this issue is closely related to the ongoing changes in climate^1^. The urgency for biodiversity assessment of modern ecosystems is compelling and the need for such results to be contextualized on a longer timescale is recognized^2^.

Conventionally, information about past variations in plant diversity is available from palynological sequences, as well as from plant fossil/subfossil time series^3^. Such records are widespread, both spatially and temporally, and therefore represent the main source of paleo-vegetational information on which estimates of past plant diversity can be inferred^4^. In general, however, palynological approaches are affected by large imbalance in taxonomic precision, which can often be limited to species aggregates, genera, families or even groups of families^3^. A new opportunity for biodiversity assessment arose more recently with the application in ecology of high throughput sequencing (HTS) to environmental DNA (eDNA)^5^. HTS of eDNA is progressively becoming a major source of data on biodiversity time series, which can be used both in long-term monitoring, and paleoecological research^6^.

HTS of eDNA is usually based on specific DNA sequences (called DNA barcodes;^7^), which generally allow to reliably discriminate species when sufficiently long and/or informative genomic regions are chosen^8^. Among the most commonly used DNA barcodes for plants, sequences of the *rbcL*, *matk* and *ITS* genes as well as the *trnH-psbA* intergenic region have been widely used^9–11^. However, additional barcodes gained increasing attention as they provide an attractive compromise between information content (i.e. level of genetic variation), length and efficiency of amplification^11,12^. Among them, the *trnL* intron of the chloroplast genome has been already widely used in diet studies based on faeces, food industry and forensic applications^13^. The short length of the *trnL* region makes it especially useful for degraded DNA, like the one extracted from poorly conserved samples and ancient specimens^14^. DNA sequencing techniques have been recently demonstrated to allow pollen grain identification in complex environmental samples through DNA metabarcoding^15,16^. The use of molecular techniques in parallel or, sometimes, alternative to conventional light microscopy pollen analyses is, therefore, becoming more and more common, and the recurrent conclusion is that results by the two different techniques complement each other. Genetic data and morphological analyses of pollen and spores complement each other due to different taxonomic resolution, often sampling spatial scales (regional for pollen vs local for DNA) and, more generally, to different taphonomic processes affecting the archiving of DNA or pollen (production, transfer and preservation processes)^17,18^. It has been proven that eDNA metabarcoding can be successfully applied to different ecosystems: from water bodies to terrestrial environments. When targeting plant biodiversity, several studies demonstrated the efficiency of estimating species content by eDNA analyses in aerial samples^15,16^, honey^19^ soil^20^ and lake sediments^21,22^. A molecular approach to reconstruct past vegetation on the ice sheet of Greenland was applied by Willerslev^23^, obtaining a record of past vegetation and invertebrates dating back to 450,000–800,000 years. Recent studies also applied eDNA metabarcoding to permafrost obtaining positive and promising results^24–26^. As low temperatures favor a good preservation of DNA molecules, frozen matrices can be ideal sources of eDNA for metabarcoding, provided no inhibitors of PCR amplification—e. g. humic acid—are present^27^, like in the case of permafrost soils. Glacier ice, in particular, is potentially a very suitable matrix for eDNA studies, as biological samples here contained are frozen, thus slowing down degradation, and glacier ice is a simple and “clean” source constituted nearly exclusively by distilled, rain water and particulate matter, thus minimizing drawbacks of DNA amplification due to inhibitors. However, to the best of our knowledge, no study has been published testing the suitability of glaciers as sources of eDNA for assessments of plant biodiversity by DNA metabarcoding. This is surprising, as glacier ice cores are potentially among the most adequate paleo-archives to investigate past variations in biodiversity for at least two major reasons. First of all, with regard to the last centuries, glaciers allow higher time resolution analyses than lakes or mires, due to the higher accumulation rate of snow (meters per year;^28^) in comparison to sediment and peat (less than mm year^-1^ to cm year^-1^;^29^). Second, studies on the pollen content of glaciers have already highlighted the high potential of these analyses in different regions of the globe. Investigations by various authors have proven that pollen grains are valuable proxies to detect seasonality in ice cores^30–36^, while other studies have shown that pollen analyses of longer glacier time series produce valid paleoecological data^37–40^. Third, glaciers have been proven to provide valuable climatic data, as they are sensitive climate indicators that adjust their mass in response to climate change^41^. Regrettably, over the last decades glaciers have already started to disappear due to the increase in atmospheric temperature^42–44^. The threat is particularly acute in the European Alps, where temperature is rising at twice the global rate^45^. This situation creates both public and scientific concern, underlining the need for a detailed investigation of glacier content. Ice core studies from the Alps already highlighted that alpine glaciers are capable of retaining useful climatic information and long time series^46–49^, setting the base for further research aimed to rescue alpine ice cores^50^. Investigating both, the biotic and abiotic component included in glaciers may provide the opportunity to directly link paleo-environmental information (obtained by eDNA analyses) to climatic information (gained from stable isotopes and geochemical proxies).

In this study, we present the results of a multi proxy case study, where we test the possibility to produce plant diversity estimates based on eDNA analyses, by the P6 loop of the plastid DNA *trnL (UAA)* intron^14^ as a marker for vascular plants, on the top surface layers of the Adamello Glacier (Italy). The results of the biotic component are discussed also in the frame of classical glaciological proxies as stable isotopes ratios (Deuterium, Oxygen), snow crystallography and glacier mass balance. This case study is the base for future research on deeper glacier ice cores encompassing longer time spans.

## Results

### Visual stratigraphy of the ice core

**Figure 1:**
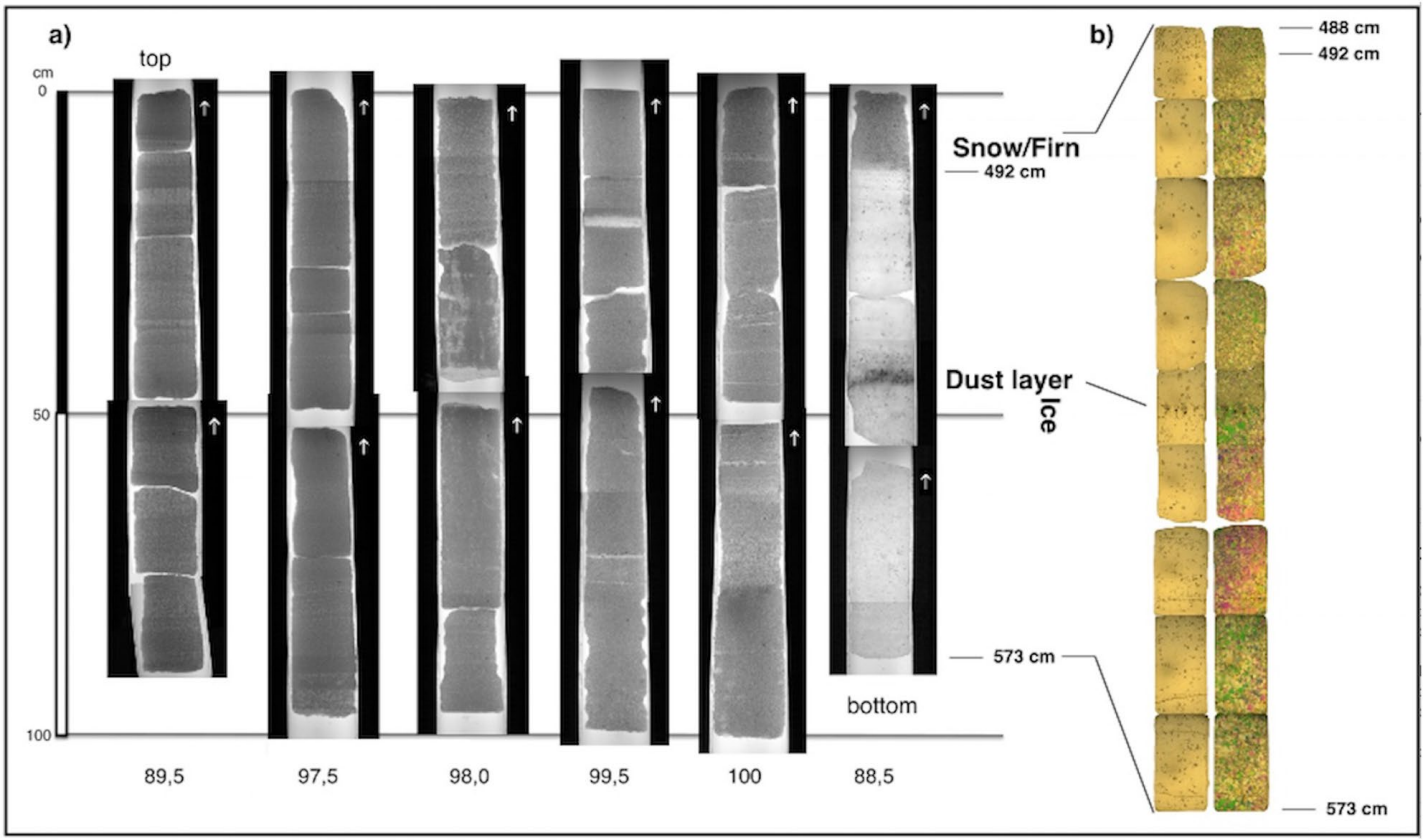
Ice core stratigraphy. (**a**) Visual stratigraphy of drilled ice core under transmitted light: below each section the total length of the single run is reported, from the top (upper left) to the bottom run (lower right). (**b**) Thin sections of the bottom part of the core with ice facies; on the left, the single polarizer images which highlight the air bubble size and density; on the right, crossed polarization filters which show ice crystal structure and size, and crystal boundaries. The sections correspond from left to right to DNA metabarcoding samples P1 to P6.

The 573 cm of ADA15 core embodied the surface snow accumulation and the upper part of ice/dense firn of the Pian di Neve, the accumulation basin of Adamello Glacier. Stratigraphically, the upper 212 cm were characterized by single snowfalls showing texture changes (particularly in grain size); the snow layers were clearly visible in the thin sections as variations in gray tones (under transmitted light) (Fig. 1a).

At 212 cm-depth, a more complex snow facies started, containing ice lenses with vertical structures, probably related to water percolation. These facies stopped at 245 cm-depth in a 5 cm-thick ice layer. This layer may be originated by surface melting, in a period with high temperature or insolation, causing percolation of water in the snowpack, and a freezing to ice within the snowpack at lower temperatures.

At a depth from 250 to 429 cm the snow facies were clearly similar to the top part, with some thin ice layers, or lenses, along the core. Some differences could be observed in snow density, mainly due to the snow grain size.

At a 492 cm-depth a transition to the ice facies was visible. After 4–5 cm of dense firn, the bottommost 81 cm of the core were represented by glacier ice, probably superimposed ice, related mostly to the complete percolation and melting of snowpack. The dense firn, in transition to ice, showed vertical and elongated and/or dendritic bubbles that represent the loss of porosity due to the formation of ice crystals. Below this small layer, there were 30 cm of ice with ice crystals of few mm in diameter and few bubbles, consistent with a strong recrystallization of the snow/firn packs after strong melting. In-fact, at 522 cm-depth, a dark layer was present, related to high accumulation of dust, representing probably a previous summer/spring glacier surface (Fig. 1b).

Below the dark level till the bottom of the core, glacier ice was present, with small crystals and more dispersed bubbles. This facies of the glacier seems to be related to the ice metamorphism, transforming snow/firn to glacier ice, even if we cannot exclude melting processes at this level (Fig. 1b).

### Stable isotopes

**Figure 2:**
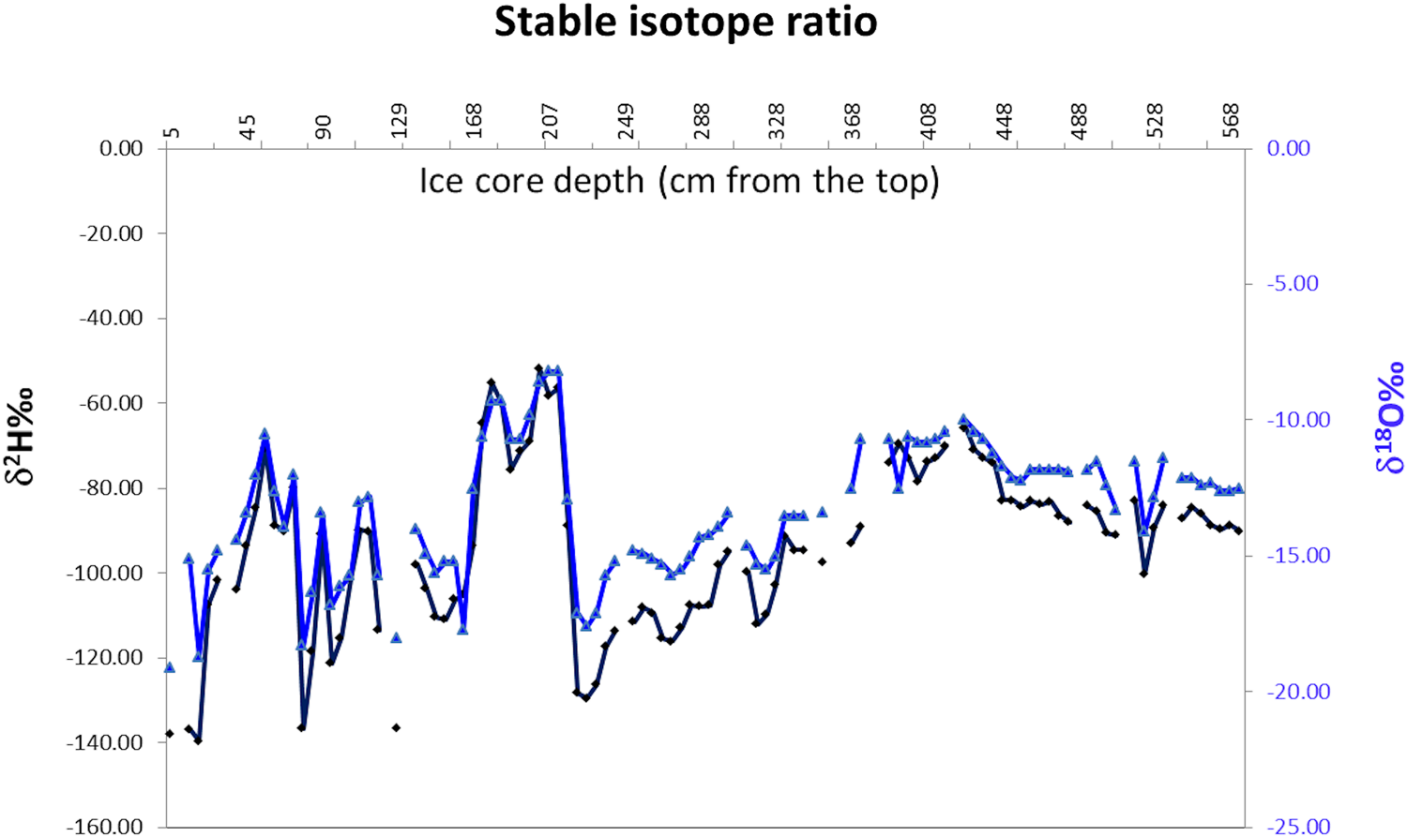
Stable isotope ratio. Values of δ^18^O‰ (in blue) and δ^2^H‰ (in black) concentration measured along the depth of the core.

Figure 2 shows the δ^2^H and δ^18^O values of melted ice. The δ^18^O values ranged between around − 17 and − 8‰ and δ^2^H values between around − 140 and − 50‰.

By evaluating the data along the ice core depth, it was possible to observe some general trends and several oscillations. Both δ^2^H and δ^18^O values had a general trend of increase from 5 to 207 cm of depth, then a drop of values at 227 cm, and again a positive trend up to 427.5 cm, followed by a negative trend down to the bottom of the core, at 567.5 cm. From the top to 207 mm of ice core depth, several significant oscillations were evident (covering up to around 70‰ for δ^2^H and more than 10‰ for δ^18^O). After the depth of 207 mm, these oscillations were much smaller.

### Microscopical pollen analyses

**Figure 3:**
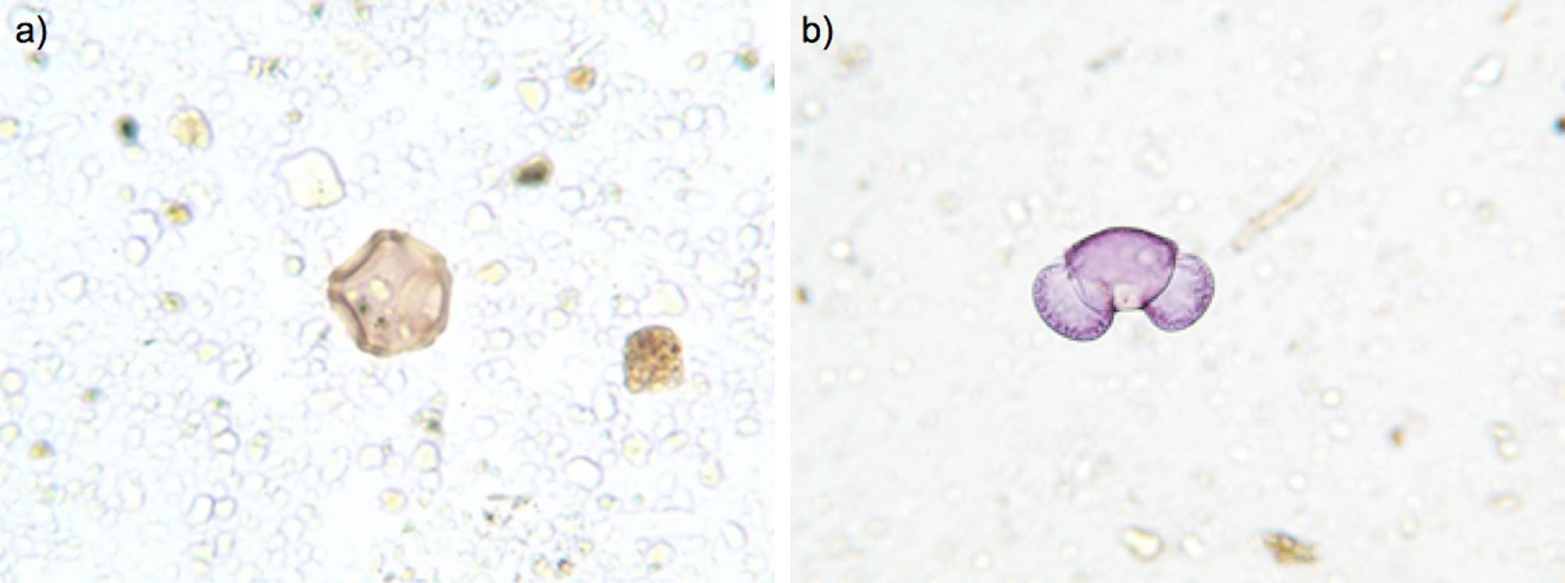
Pollen grains retrieved from the ice core and analysed by light microscopy. Pollen grains of *Alnus* (**a**), *Ambrosia* (**b**), *Artemisia* (**c**) and *Pinus* (**d**).

Palynological analyses on three random samples confirmed the presence of pollen grains within the ice core (Fig. 3; Table 1). In addition to pollen, various other debris were present in the samples (Fig. 3), some likely of vegetal origin. The 14 identified pollen taxa belonged to a total of 10 families, with the families of Cupressaceae and Taxaceae, characterized by pollen grains with the same morphological characteristics, being the most represented. This winter pollen occurs in large amounts in the sample Bio-5, at a depth of 50 cm from the surface, where the core should represent the early winter precipitation of 2015. The other pollen taxa, dominated by Poaceae, are all compatible with the vegetation of the catchment area. Four of the 10 families (Brassicaceae, Malvaceae, Polygonaceae and Ranunculaceae) were identified exclusively by microscopical analyses and not by metabarcoding (see below).

**Table 1:** Taxonomic assignment and counts from palynological analyses of three random 10-cm samples from Adamello glacier core.

	Sample max depth (cm)		
	50	114	182
*Alnus*	1	0	0
Cupressaceae/Taxaceae	61	1	2
*Pinus*	1	2	4
*Corylus*	2	0	0
Poaceae	7	8	5
Urticaceae	0	1	2
Polygonaceae	0	1	1
Ranunculaceae	0	2	0
*Ostrya carpinifolia*	0	0	1
*Ulmus*	0	0	1
*Tilia*	0	0	1
*Quercus*	3	2	1
Brassicaceae	0	1	0
*Carpinus betulus*	0	0	1
Unidentified	4	8	7

Figures in the table are the number of pollen grains detected for each taxon in the corresponding sample. Column headings show the relative depth of samples from the surface firn layer.

### Environmental DNA sequencing and data analysis

Raw reads from lllumina MiSeq showed a very high accuracy, with an average Phred Quality Score of 40.9, corresponding to an Inferred Base Call Accuracy > 99.99%, which is a probability of incorrect base call of 1:10,000^51^ (Illumina Technical note: https://www.illumina.com/documents/products/technotes/technote_Q-Scores.pdf).

Sequencing of firn eDNA amplicons generated a total of 190,028 raw reads, 158,724 of which passed the quality filtering. After removal of singletons and chimeras, sequencing depth was from 16,948 to 37,471 reads per sample (Supplementary Table S1). Samples were subjected to rarefaction (without replacement) at 16,948 reads per sample, corresponding to the minimum number of reads per sample (sample PI2). Reads were clustered in a total of 2339 OTUs, with a maximum of 1047 OTUs per sample (corresponding to the sample PI5). Sequencing results are detailed in the Supplementary Table S1.

The obtained dataset was the basis for the following taxonomic assignment.

### Taxonomic assignment of environmental DNA sequences

**Figure 4:**
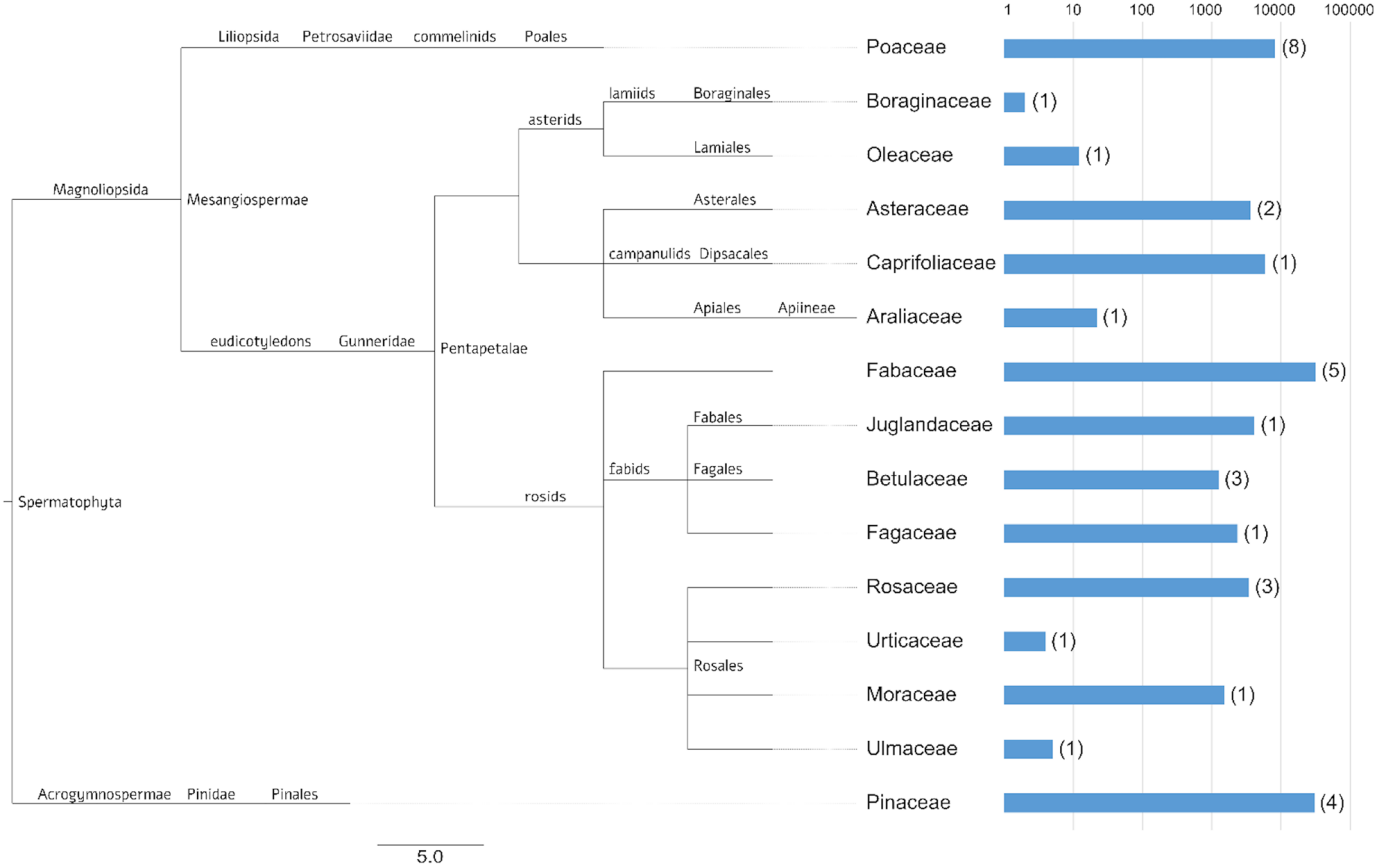
Taxonomy of plant families corresponding to the OTUs identified by metabarcoding of the trn-L marker on firn core samples from Adamello glacier. Bar lengths represent in logarithmic scale (base 10) the number of reads assigned to a specific family. Numbers above each bar are the number of OTUs identified. Tree was visualized with FigTree software (http://tree.bio.ed.ac.uk/software/figtree/).

The data analysis of OTUs was performed against the local reference database at a 99% similarity, resulting in 2164 OTUs (92.5%) taxonomically assigned to a single plant taxa. Overall, Fabaceae, Pinaceae and Poaceae were the most represented plant families, representing 69% of the total reads with a taxonomic assignment in the reference database. The taxonomic distribution of the OTUs spanned the whole spermatophyta clade from gymnosperms to angiosperms (Fig. 4), representing a total of 10 orders. In gymnosperms only the Pinaceae family was represented, while in angiosperms a higher number of families known to encompass species with anemophilous pollination was identified, spanning both monocotyledons and dicotyledons. In dicotyledons, both rosids and fabids clades were represented. While Pinaceae and Poaceae were identified also by light microscopy analyses, Fabaceae were identified exclusively by DNA metabarcoding. The two families with the highest numbers of reads, Pinaceae and Fabaceae, encompassed alone > 60% of the reads that could be identified. While in general the number of reads was often higher in families with higher numbers of taxa (see, e.g. Poaceae, Fabaceae and Pinaceae), the Caprifoliaceae were represented by only one genus (*Lonicera*; Fig. 4; Supplementary Table S2). In the case of sequences identified as *Ficus carica*, a curated BlastN search was carried out to the NCBI nucleotide database, resulting in the high-confidence identification of the sequences as *Broussonetia papyrifera* (L.) Vent., an invasive species of Asian origin.

**Figure 5:**
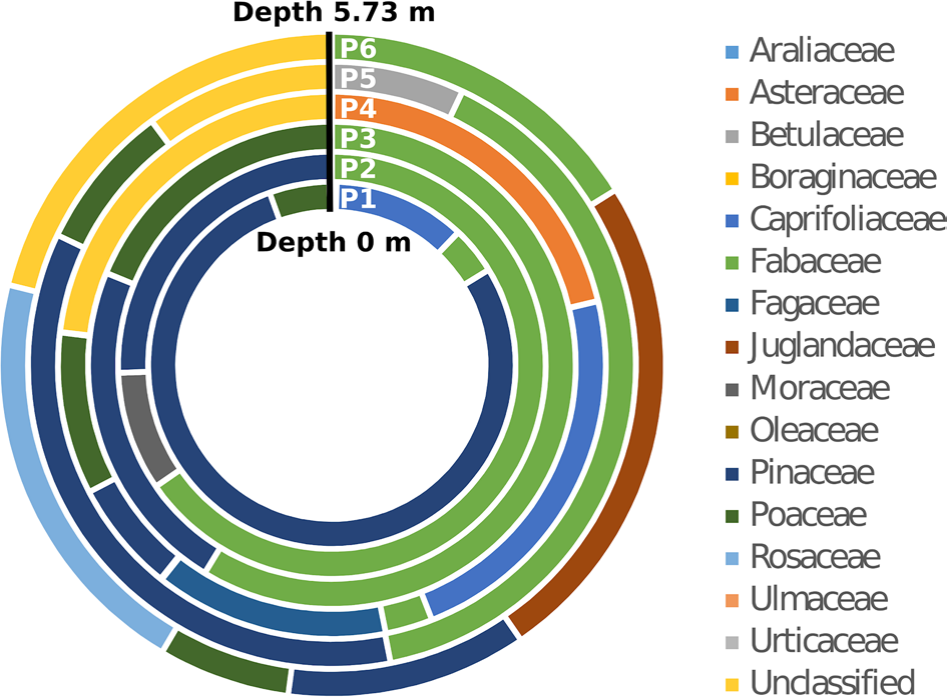
Doughnut chart showing plant diversity at family level at the six depths of the firn core. Relative abundances of taxa are symbolized by the width of the corresponding arch. Unclassified represents plant taxa with no blast hit in the reference database.

The overall diversity is shown in the doughnut chart of Fig. 5, where each sample is symbolized by a circle, with the arch width proportional to the frequency of plant families. Plant biodiversity is clearly changing from one sample to another, supporting the estimated different flowering periods and years, as suggested by conventional light microscopy pollen analyses too.

**Figure 6:**
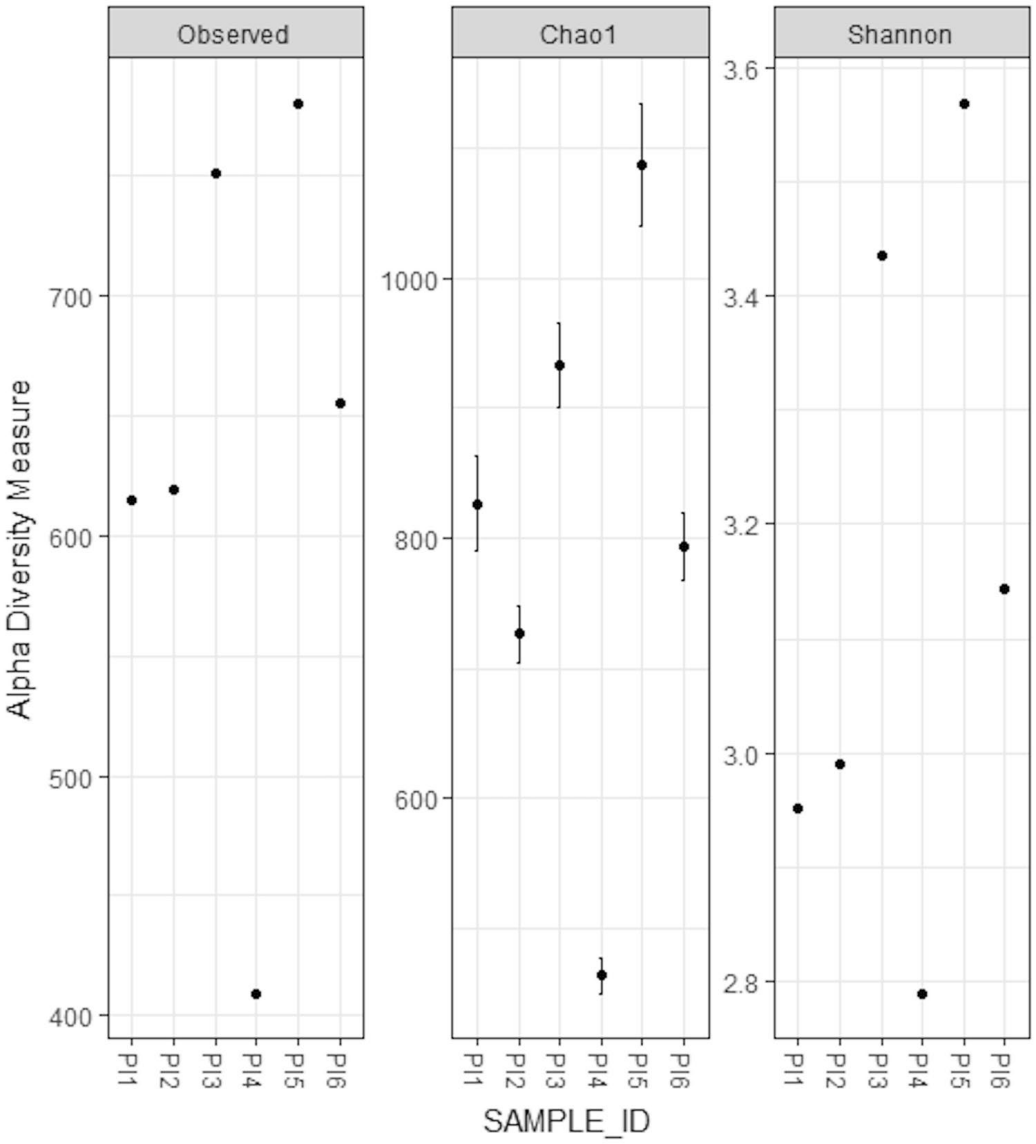
Alpha diversity by sample. The number of observed OTUs has been plotted for each sample, Chao1 and Shannon entropy diversity indices are reported. The bars are the standard errors as defined for the Chao1 model for estimating richness.

When analyzed by Chao1 and Shannon diversity indices, the alpha diversity estimated for the samples resulted in a large range of values. Both indices assigned a very high alpha diversity to samples PI3 and PI5 and low alpha diversity for sample PI4 (Fig. 6).

The overall results (Supplementary Table S2) described 36 different plant taxa, with different taxonomic resolution. eDNA metabarcoding, associated with high throughput sequencing, assigned taxonomy mostly to the species level (21; 58.3%). In only one case the taxonomy was assigned to more than one species of the same genus (2.8%); in three cases to the genus level (8.3%) and in other three to more than one genus in the same family (8.3%). Assignments at the family level covered 8 cases (22.2%).

## Discussion

In this study we tested whether metabarcoding of eDNA using the P6 loop of the plastid DNA *trnL (UAA)* intron^14^ could be used as a proxy of vascular plant diversity when analyzing surface layers of the largest Italian glacier, the Adamello Glacier. The high sequence diversity of the trnL p6 loop region^14^ allowed us to use a stringent cutoff of 99% sequence identity for the taxonomic assignment of the OTUs, higher than the 97% adopted by similar studies (e.g.^15,52^). Despite the reduced length of the barcode marker sequence (138–152 bp, depending on the taxa), such approach resulted in a higher accuracy of the taxonomic assignment, as compared with that achievable by conventional palynology^3^. As expected, the two methods did not provide fully overlapping taxonomic representations, as indicated by the method-specific identification of some of the taxa. Palynological analyses by conventional light microscopy, however, were performed on a low number of samples, since they aimed mainly at confirming the presence of pollen rather than to compare the two approaches, as this has been extensively done in previous studies (reviewed in^53^). Additionally, as shown by the conventional light microscopy analysis, glacier ice trapped, besides pollen grains, also plant remains, likely blown by wind during storms^54^, which can further contribute to assess plant species composition of the surrounding area, and to consider palynological and molecular analyses as complementary tools for biodiversity assessment^53^. Also the taphonomic processes acting on the pollen grains in the ice archive can contribute, at least in part, to the observed differences between the taxa identified by the two methods, although taphonomy of eDNA from glaciers remains largely to be investigated. Compared to eDNA from lake sediments, where extracellular DNA has been suggested to constitute the major component^18^, extracellular DNA in the ice samples of a temperate glacier as Adamello is expected to be a minor fraction for the following reasons: (1) repeated cycles of freezing and thawing can mechanically degrade exposed DNA through cryolysis^55^; (2) extracellular DNA in the ice cannot complex with soil particles, which have been demonstrated to protect it from nuclease action^17,56^; (3) If dissolved in melting water, despite the low temperature, the DNA is exposed to hydrolysis, due to the low ionic strength and to physical degradation due to UV exposure^18,57,58^; (4) once dissolved in melting water extracellular DNA can be diluted below the detection limit or completely removed through percolation. As the visual stratigraphy and the stable isotope analyses we carried out suggest significant processes of meltwater percolation and refreezing of the ice core, we suggest that the majority of DNA analyzed by metabarcoding is of intracellular origin, both from pollen grain and plant debris. The lack of Cupressaceae and Taxaceae sequences from the metabarcoding record is in line with this scenario, as for these families, the exine layer of pollen walls is known to be very thin and easily broken^59,60^, causing the release of the pollen protoplast due to pollen bursting^61^. Once in the low ionic strength meltwater, the protoplasts can undergo rupture by hypo-osmotic stress and release their DNA extracellularly. Analogous taphonomic processes may differentially affect DNA preservation as well as transport processes in different species, possibly causing a partially biased representation of the plant biodiversity of the palynological catchment area.

In this study, eDNA was analyzed at six depth ranges, for a total length of about 6 m. The marked variations among ice core fractions detected by both the palynological and the isotope ratio analyses suggested that the investigated ice layers correspond to multiple seasons, possibly in the range between 4 to 6 years. The progressive reduction of signal in the stable isotope ratio data suggested a possible modification in the solid precipitation after the deposition, by processes of meltwater percolation and refreezing, which can reduce seasonal isotopic signals, induce isotopic enrichment and introduce time gaps^62^. This could be the case for the sampled ice core described by these analyses; deeper drilling in additional locations will allow to ascertain whether melt-affected ice is a general feature of the Adamello glacier or if it is a local peculiarity at the drilling site. Due to these processes, chemical signals are generally susceptible to various modifications which can affect the quality and quantity of information when analyzed on temperate glaciers. Palynological analyses, on the contrary, demonstrated to provide valuable information even when in temperate glaciers, where low latitude and climatic conditions do not guarantee freezing temperatures all year long^31^, due to the stability of these biological particles in the ice layers.

In general, the taxonomic representation resulting from eDNA metabarcoding was surprisingly broad, reflecting many plant species from the families of Betulaceae, Fagaceae, Oleaceae, Pinaceae, Poaceae and Urticaceae, typical of the alpine habitats at different elevations within the palynological catchment area of the Adamello glacier^63^. In our study, species accumulation curve reached saturation (see Supplementary Fig. S1), indicating the exhaustive sampling of almost all species present in DNA samples. Moreover, taxonomic assignment after eDNA metabarcoding was performed against a custom-made reference database^16^, ensuring data quality by a robust set of reference DNA sequences, as suggested by^64^. The only OTU identified by eDNA metabarcoding which could not directly be explained by the custom reference database used in this study, was the OTU initially identified as *Ficus carica*, a mediterranean species, which does not spontaneously grow in the immediate proximity of the glacier. Manual curation of the homology search, however, easily fixed the representativeness problem and identified the taxon as *Broussonetia papyrifera*, an invasive species, occasionally naturalized, and present in several Italian regions, including Trentino, Alto-Adige and Lombardy, which surround the Adamello glacier^65^. Recently, *B. papyrifera* has been recognized as a source of pollinoses in Asia^66^, and it is currently object of monitoring by PollNet, the Italian network of aerobiological monitoring centres (http://www.snpambiente.it/2019/05/10/primo-studio-in-italia-sulla-distribuzione-del-polline-di-broussonetia-papyrifera/ downloaded at February 28, 2020). The small size of *B. papyrifera* pollen, closely similar to Urticaceae pollen, explains its high dispersal ability. The detection of *B. papyrifera* pollen on the Adamello glacier indicates the high potential of glacier ice as a proxy of vascular plant diversity of the catchment area, including even alien/invasive species.

Nowadays the integration of the DNA metabarcoding and the morphological approaches represent the best way to investigate the major part of the ecological sites^67^. Despite the accuracy of DNA metabarcoding analysis is strictly related to the reference database used and its quantification capability is still under study^68^, the advantages of the metabarcoding approach (overcoming the morphological approach limits, low cost and low time consumption) make it a fundamental method for ecological survey^6,53^.

In conclusion, we demonstrated for the first time that a biological signal is archived in the accumulation basin of the Adamello glacier and that eDNA metabarcoding is a feasible technique when applied to eDNA of firn and ice of Alpine glaciers. Moreover, eDNA metabarcoding gives a high-ranking taxonomic resolution to pollen signal, capable of disclosing plant biodiversity of the palynological catchment area, estimated by the traces of pollen and plant remains archived in this Alpine temperate glacier. The potential of sampling glacier DNA with eDNA metabarcoding will need to be further investigated in other glaciers and to different depths, to fully exploit the wealth of information still contained in these important archives of natural plant biodiversity. The relevance of this information on heavily endangered sites could improve existing knowledge on plant biodiversity changes during the past in large catchment areas and on the link of these changes to distinct climatic conditions, leading to results that could be applied on estimates of climate change effects on the future vegetational composition of the ecosystem.

## Methods

### Study site

**Figure 7:**
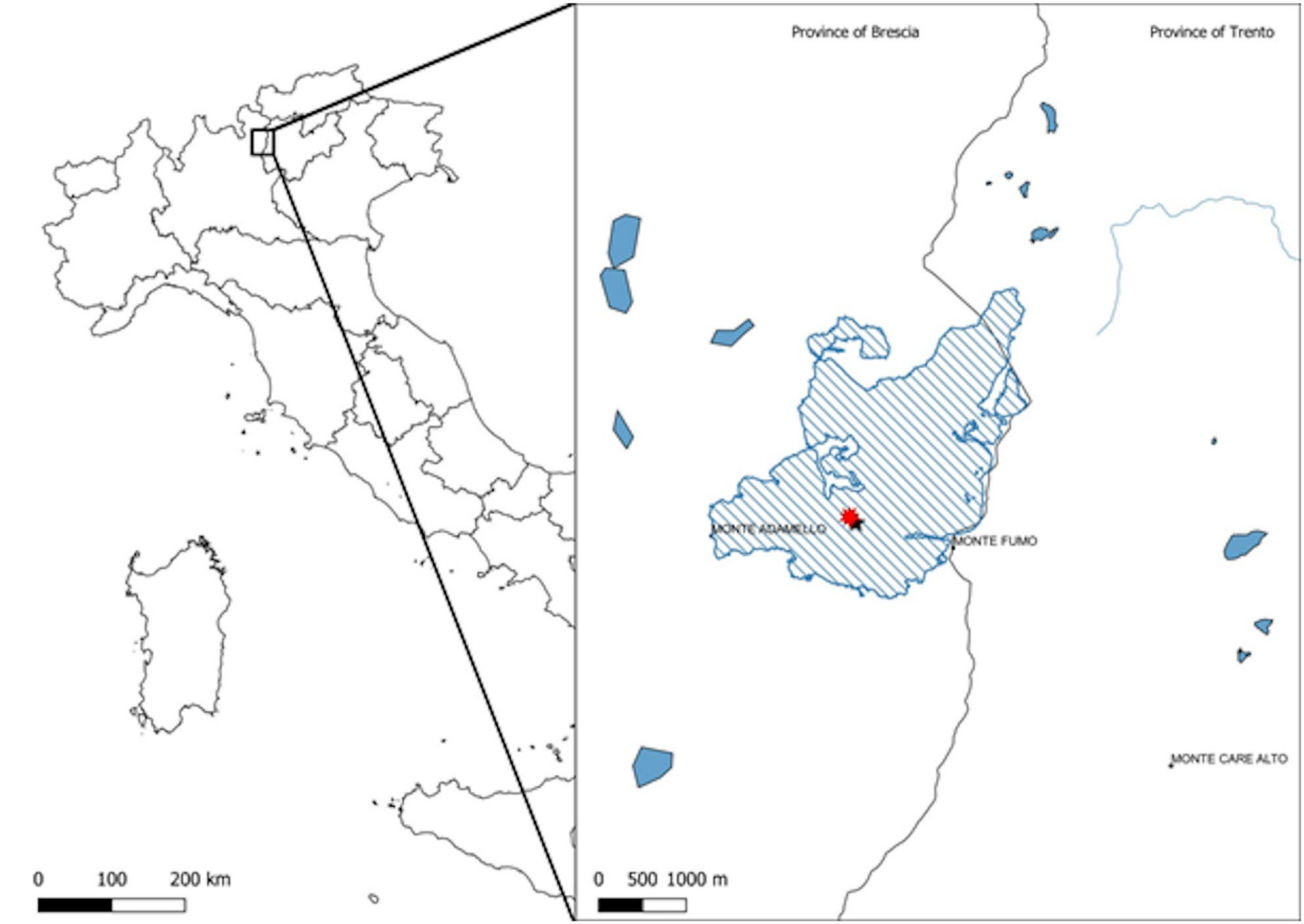
Study area location in Italy, with the Adamello glacier (in blue stripes) and drilling site (red dot).

The drilling site (coordinates UTMWGS84, E 617616–N 5111704) is located on Pian di Neve, the vast plateau that represents the accumulation basin of the Adamello Glacier which is situated between Brescia and Trento provinces in the Adamello-Presanella group (Fig. 7). Recent seismic geophysical surveys estimated the maximum thickness of the glacier (270 m;^69^) and the main directions of the glacial flows.

Adamello Glacier has been classified as a unique glacial unit^70^ and comprises the six units in which the glacier was divided in the Inventory of the Italian Glaciological Committee^71^: Miller Superiore (CGI n.600), Corno Salarno (CGI n.603), Salarno (CGI n. 604), Adamello or Pian di Neve (CGI n.608), Adamè (CGI n.609) and Mandrone (CGI n. 639). Considered in its entirety, Adamello Glacier can be classified as a Scandinavian summit glacier^72,73^. More precisely, it should be classified as a plateau glacier with radial tongues.

Pian di Neve is a large accumulation area from which the different tongues originate, the main of which is channeled towards the Val Genova (Mandrone Glacier). Pian di Neve plateau receives the contribution of other glacial flows originating from both the north (Corno Bianco) and the south-west (Corno di Miller, Corno di Salarno and Cornetto di Salarno).

Adamello Glacier is fed by direct snowfall. In recent years the snow limit has often been placed at very high altitudes and the entire central glacier sector, including Pian di Neve, has been found in ablation conditions. The accumulation areas are reducing their size and are now concentrated in correspondence with the highest cirques, both in the eastern and western glacier sectors. Moreover, the open morphology reduces the shading effect due to the rocky ridges that delimit it.

Adamello Glacier is currently the largest glacier in the Italian Alps, with an area of 15.5 km^2^ in 2015 (data downloadable at https://webgis.provincia.tn.it, update 2015 by Casarotto, C. and Trenti, A., accessed 28 April 2020). The total area of the glacier exceeded 30 km^2^ in 1864^74^; it was 17.97 km^2^ between the years 1959–1961^70^, 18 km^2^ in 1987^75^, 17.63 km^2^ in 1997^76^ and 16.44 km^2^ in 2011 (orthophotos by Autonomous Province of Trento).

Mandrone Glacier has retreated about 2 km from 1820, at the end of the Little Ice Age. From 1984 to 2014 the glacier front retreated 243 m, with an average annual shrinkage of about 8 m (data from the Italian Glaciological Committee). Following this retreatment, the Adamello Glacier is fragmenting, leading to the isolation of glaciers that remain localized in small glacial cirques.

As regards the only Trentino sector, the current area (2013) is 78.5 ha. In 1987 it was 141.6 ha, while in 2003 it was 123.8 ha. The annual percentage reduction was 0.78% in the period 1987–2003 and then increased to 3.66% in the decade 2003–2013. These data show that in the last period the annual reduction rate was 4 times higher than in the previous period 1987–2003^77^.

### Ice core processing and visual stratigraphy

A 573 cm long core was drilled through the Adamello glacier (Trentino, Italy) in March 2015, by a mechanical drill system (SIPRE type, USA), with a core diameter of 100 mm and length of approximately 60 cm for each section. The sampled core was cut at Eurocold Lab, University of Milano Bicocca, in 5 cm sections for the stable isotope ratio analysis, and 10 cm sections for environmental DNA (eDNA) analyses. Samples were stored at − 20 °C. Thin sections, 1 mm thick, were cut from the ice core and placed between two crossed polarization filters. Size and orientation of the ice crystals were evaluated by the different color of the crystals, due to the polarization of the light.

### Stable isotope ratio

Samples were thawed at room temperature in hermetically sealed tubes. As described in previous papers^78,79^, the ratio ^18^O/^16^O of water was analysed using an isotope ratio mass spectrometer (Isoprime, Manchester, UK) interfaced with an on-line automatic system that allows CO_2_/H_2_O equilibration (Multiflow, Isoprime, Manchester, UK). For the analysis of ratio ^2^H/^1^H of water, 200 μl of water together with Hokko bead platinum catalyst (Isoprime) were introduced into a reaction vessel, which was then attached to the online automatic equilibration system and filled with He containing 10% of H_2_. The ratios ^18^O/^16^O and ^2^H/^1^H were expressed as delta per thousand (δ^18^O and δ^2^H‰), computed by comparing the isotope ratios of the sample to those of the international reference standard V-SMOW (Vienna-Standard Mean Ocean Water, IAEA-International Atomic Energy Agency, Vienna, Austria) on a scale normalized by assigning the consensus value of − 55.5‰ to V-SLAP (Standard Light Antarctic Precipitation, water, IAEA). For calibration, working in-house standards (tap water) calibrated against V-SMOW and V-SLAP were used. Analytical uncertainty (1 standard deviation) of δ^2^H and δ^18^O analysis was < 2 and < 0.2 ‰, respectively.

### Light microscopy pollen analysis

In order to verify the presence of pollen grains within the ice core, random samples, about 35 ml each were concentrated and processed with acetolysis^80^. Afterwards, pollen was mounted on slides, coloured with basic fuchsin and analysed by light microscopy (400 × magnification) for the identification of pollen grains by means of standard identification keys^81^, and comparison to a local pollen collection (Fondazione Mach pollen reference collection) and to pollen atlas (http://pollini.iasma.it/polimage;^82^).

### Environmental DNA sample processing and extraction

Ten cm-long samples were thawed in sterile glass containers in a refrigerator for 24 h and subsampled for eDNA, as to be equally represented in the resulting composite samples. The six composite samples covered the entire length of the ice core, representing the following relative depth from the top: 0–172 cm (PI1); 173–275 cm (PI2); 276–449 cm (PI3); 450–489 cm (PI4); 490–509 cm (PI5); 510–573 cm (PI6).

A 36 ml aliquot of each composite sample, together with a negative control, was individually concentrated (Concentrator Plus, Eppendorf, Hamburg, Germany) to a final volume of 100 µl (6 to 7 h).

DNA extraction protocols were applied under a high performance Class II biosafety cabinet in a non-invasive DNA laboratory, free from contaminant small DNAs. Details on the procedures and controls used to prevent and identify contaminations are provided in Supplementary Methods. An automated high throughput (HTP), magnetic-bead based isolation of genomic DNA was performed by NucleoMag Plant, (Macherey–Nagel, Düren, Germany) and KingFisher Flex platform (Thermo Scientific, MA USA). Cell lysis was promoted by mechanical disruption (Mixer Mill MM200, Retsch GmbH, Germany). In order to control contamination during extraction, samples were loaded in plates, following a design where empty wells surrounded each sample (see Supplementary methods). The final elution volume for all samples was 100 µl.

### Library preparation and sequencing

Total eDNA was subjected to PCR amplification by targeting a ~ 140-bp fragment of the P6 loop region within the chloroplast trnL (UAA) intron (Taberlet et al. 2007). The specific chloroplast DNA primers set *c* pos. 49,325 (5′-CGAAATCGGTAGACGCTACG-3′) and *h* pos. 49,466 (5′-CCATTGAGTCTCTGCACCTATC-3′) were used, fitted with overhang Illumina adapters.

Each sample (negative controls and blank included) was amplified by a 1^st^ round of PCR using 50 µl reaction with 1 µM of each primer. More in detail, 10 µl of 5 × GO Taq Flexi Buffer and 2 µl forward and reverse primers, were used in combination with 5 µl of template DNA (5 ng/µl), 6 µl MgCl_2_ 25 mM, 0.25 µl dNTP 40 mM and 0.25 µl GO Taq Flexi [5U/µl]. PCR reactions were executed in triplicates by a Veriti 96 well thermal cycler (Applied Biosystems, Foster City, CA, USA) at the following cycling conditions: initial denaturation step at 95 °C for 2 min (one cycle); 40 cycles at 95 °C for 15 s, 52 °C for 15 s, 72 °C for 30 s; final extension step at 72 °C for 5 min (1 cycle).

The amplification products in triplicate were pooled, subsequently the amplicons library construction and the high throughput sequencing were performed at the FEM Sequencing Platform using an Illumina MiSeq (MiSeq Control Software 2.5.0.5 and Real-Time Analysis software 1.18.54.0).

### Bioinformatics analysis

Raw reads were processed with the MICrobial Community Analysis software MICCA v. 1.7.2^83^ by setting the OTUs clustering at the 99% sequence similarity level and 75% query coverage, and, subsequently, OTU classification was performed against the BioAir reference database^16^. Chao1 and Shannon indices were estimated to calculate alpha diversity in terms of OTUs richness and diversity. Taxonomic tree for the OTUs was reconstructed using the NCBI taxonomy and visualized in FigTree v. 1.4.4. Manual curation of samples was carried out by BLAST analysis of selected sequences to the Nucleotide Database of the National Center for Biotechnology Information (NCBI, 8600 Rockville Pike, Bethesda MD, 20894 USA).

## Supplementary information

Supplementary Information.

Supplementary Information.

## Data Availability

Raw sequences were deposited in the European Nucleotide Archive ENA-EMBL, under the accession number PRJEB28043.
