# Supplementary material for: A pilot study of eDNA metabarcoding to estimate plant biodiversity by an alpine glacier core (Adamello glacier, North Italy)
Source: Sci Rep. 2021 Jan 13;11:1208. doi: 10.1038/s41598-020-79738-5 (PMC7807053; doi:10.1038/s41598-020-79738-5)

Supplementary information

A pilot study of eDNA metabarcoding to estimate plant biodiversity by an alpine glacier core (Adamello glacier, North Italy)

Claudio Varotto[1], Massimo Pindo[1], Elena Bertoni[2], Christian Casarotto[3], Federica Camin[1], Matteo Girardi[1], Valter Maggi[4], Antonella Cristofori[1,*]

[1]Research and Innovation Centre, Fondazione Edmund Mach, San Michele all'Adige (TN), Italy

[2]Uppsala University, Uppsala, Sweden

[3]Geology department, Science Museum (MUSE), Trento, Italy

[4]Earth and Environmental Sciences, University of Milano Bicocca, Milano, Italy

*Corresponding author: antonella.cristofori@fmach.it

Supplementary TableS1. Sequencing results per taxa of the six samples (P1-P6, referring to diferent depths of the ice core), in terms of filtered reads. Total # of raw reads and OTUs per sample is also reported.

Taxa	PI1	PI2	PI3	PI4	PI5	PI6
Agrostis capillaris	0	0	0	0	0	2
Asteraceae	0	0	0	3327	0	0
Betula pendula	88	0	0	0	0	0
Betulaceae	0	0	0	0	12	0
Carpinus/Corylus/Ostrya	0	1	1	0	2384	1
Elymus caninus/E. repens	0	0	3831	8	0	0
Eritrichium nanum	0	0	0	4	0	0
F. ornus/F. excelsior/Olea europaea/Syringa vulgaris	2	0	0	11	0	0
Fabaceae	1128	10470	10385	381	12068	4476
Fagaceae	0	1	0	2697	0	0
Festuca rubra/Trisetum spicatum/others	0	0	5	0	0	0
Ficus carica	0	1370	0	0	0	0
Hedera helix	27	0	0	4	0	0
Juglans regia	0	0	2	0	0	6612
Laburnum anagyroides	0	0	0	36	34	0
Larix decidua	0	1	0	0	2	10
Lonicera *	3209	0	0	4418	0	0
Picea abies	2897	0	0	1	224	12
Pinaceae	220	36	52	19	137	35
Pinus mugo	18715	4115	5357	1312	12161	3197
Pisum sativum	0	0	1	0	17	0
Poa annua	0	0	0	0	0	13
Poa badensis	0	0	0	0	0	1700
Poa pratensis	0	0	513	1887	7	0
Poaceae	0	0	10	13	2152	12
Prunus armeniaca	0	0	0	0	0	3891
Rosaceae	0	0	0	0	0	4
Rubus caesius	0	0	0	0	0	1666
Setaria verticillata	1407	0	0	0	464	0
Taraxacum *	0	0	0	810	0	1
Trigonella caerulea	0	0	3609	0	1846	0
Ulmus glabra	0	0	0	4	0	0
Urticaceae	0	3	0	0	0	0
Vicia cracca	0	0	3	1	17	0
Unclassified	58	6	8	4629	3635	5882
Total # of filtered reads	27751	16003	23777	19562	35160	27514
Total # of raw reads	33289	31735	28523	23205	40091	33185
Total # of OTUs	735	647	852	452	1047	793

*Deposited sequence could not be verified by GeneBank, because it lacks annotation

Supplementary Table S2. List of taxa (different taxonomic ranks) with metabarcoding (eDNA) and conventional light microscopy (Morphology) hits.

Growth form	Above-family	Family	Above-genus	Genus	Above-species	Species	Morphology	eDNA
Herbs		Asteraceae		*Taraxacum* *				v
		Asteraceae					v	v
		Fabaceae				*Pisum sativum*		v
		Fabaceae				*Trigonella caerulea*		v
		Fabaceae				*Vicia cracca*		v
		Poaceae	Festuca rubra/Trisetum spicatum/others					v
		Poaceae			*Elymus caninus/E. repens*			v
		Poaceae				*Agrostis capillaris*		v
		Poaceae				*Poa annua*		v
		Poaceae				*Poa badensis*		v
		Poaceae				*Poa pratensis*		v
		Poaceae				*Setaria verticillata*		v
		Poaceae					v	v
		Urticaceae					v	v
		Fabaceae						v
Shrubs		Araliaceae				*Hedera helix*		v
		Boraginaceae				*Eritrichium nanum*		v
		Caprifoliaceae		*Lonicera* *				v
		Pinaceae				*Pinus mugo*		v
		Rosaceae				*Rubus caesius*		v
Trees		Betulaceae	*Carpinus_betulus/Corylus avellana/Ostrya*					v

			carpinifolia					
		Betulaceae		*Alnus* sp.			v	
		Betulaceae				*Betula pendula*		v
		Betulaceae		*Betula* sp.			v	
		Betulaceae				*Alnus viridis*	v	
		Betulaceae				*Carpinus betulus*	v	
		Fagaceae	*Quercus ilex* T.				v	
		Fagaceae	*Quercus robur* T.				v	
		Fagaceae		*Fagus* sp.			v	
		Fagaceae				*Castanea sativa*	v	
		Fagaceae						v
		Juglandaceae		*Juglans* sp.			v	
		Juglandaceae				*Juglans regia*		v
		Malvaceae		*Tilia* sp.			v	
		Moraceae				*Broussonetia papyrifera*		v
		Myrtaceae		*Eucalyptus* sp.			v	
		Oleaceae	*Fraxinus_excelsior/F. ornus/Olea europaea/Syringa vulgaris*					v
		Oleaceae		*Olea* sp.			v	
		Oleaceae				*Fraxinus excelsior*	v	
		Oleaceae				*Fraxinus ornus*	v	
		Pinaceae		*Abies* sp.			v	
		Pinaceae		*Larix* sp.			v	
		Pinaceae		*Picea* sp.			v	
		Pinaceae				*Larix decidua*		v
		Pinaceae				*Picea abies*		v
		Pinaceae				*Pinus cembra*	v	
		Pinaceae						v

		Salicaceae		*Populus* sp.			v	
		Ulmaceae		*Ulmus* sp.			v	
		Ulmaceae				*Ulmus glabra*		v
	Cupressaceae/Taxaceae						v	
		Betulaceae						v
		Fabaceae				*Laburnum anagyroides*		v
		Oleaceae					v	
		Rosaceae				*Prunus armeniaca*		v
		Rosaceae						v

*Deposited sequence could not be verified by GeneBank, because it lacks annotation.

Supplementary Figure S1. OTU accumulation curves for each of the six samples, expressed as the number of OTUs by the number of reads from sequencing.

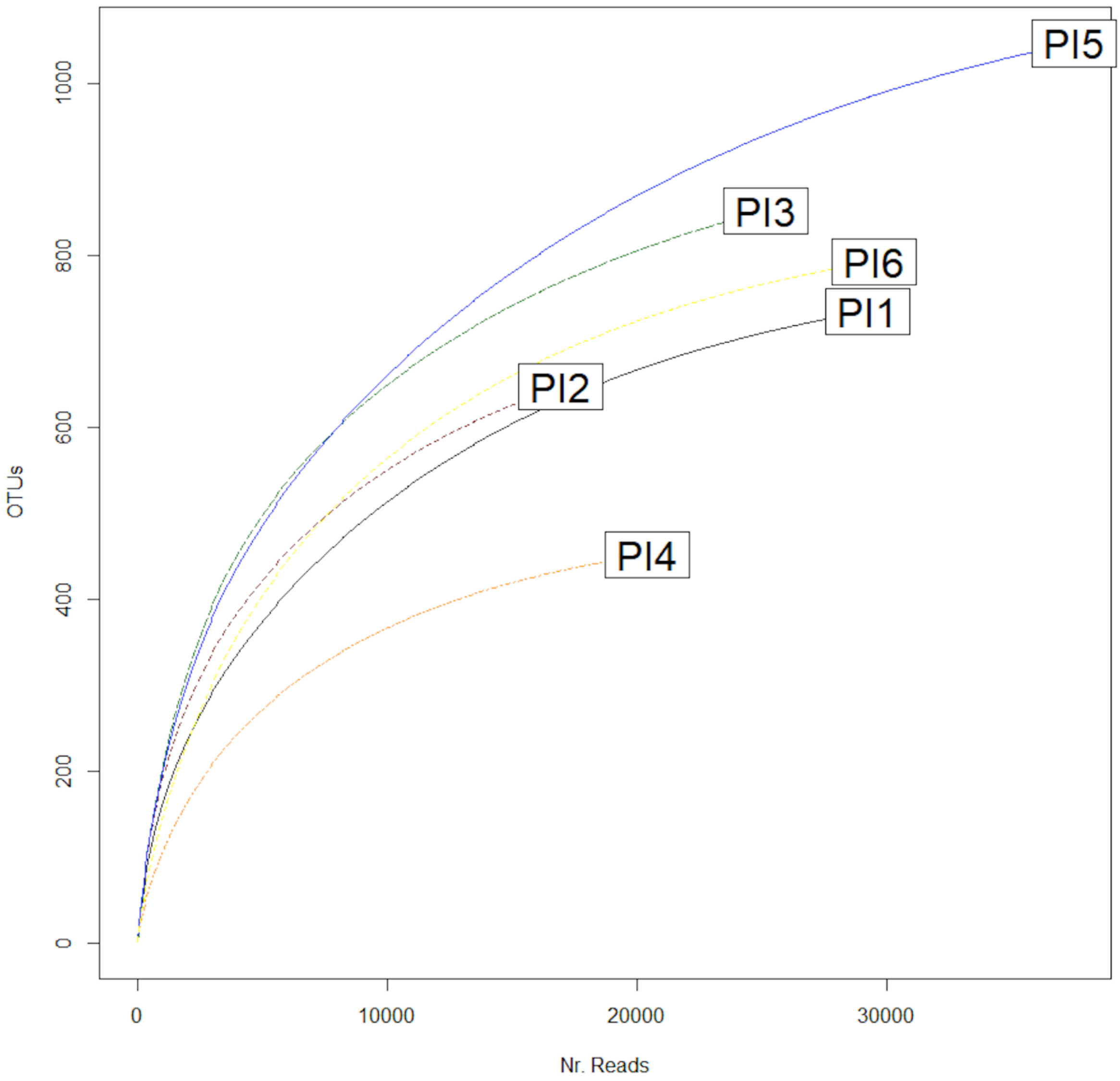

Supplement: Supplementary file 1 — Supplementary Information. [file 41598_2020_79738_MOESM1_ESM.pdf]
