# Supplementary material for: A pilot study of eDNA metabarcoding to estimate plant biodiversity by an alpine glacier core (Adamello glacier, North Italy)
Source: Sci Rep. 2021 Jan 13;11:1208. doi: 10.1038/s41598-020-79738-5 (PMC7807053; doi:10.1038/s41598-020-79738-5)
Supplement: Supplementary file 2 — Supplementary Information. [file 41598_2020_79738_MOESM2_ESM.pdf]

Supplementary Methods

A pilot study of eDNA metabarcoding to estimate plant biodiversity by an alpine glacier core (Adamello glacier, North Italy)

Claudio Varotto[1], Massimo Pindo[1], Elena Bertoni[2], Christian Casarotto, Federica Camin[1], Matteo Girardi[1], Valter Maggi[4], Antonella Cristofori[1,*]

[1]Research and Innovation Centre, Fondazione Edmund Mach, San Michele all'Adige (TN), Italy
[2]Uppsala University, Uppsala, Sweden
[3]Geology department, Science Museum (MUSE), Trento, Italy
[4]Earth and Environmental Sciences, University of Milano Bicocca, Milano, Italy

*Corresponding author: antonella.cristofori@fmach.it

Control of DNA contamination

Experimental details in the environmental DNA sample processing and extraction

Experimental procedures were applied in order to monitor and minimise DNA contamination. Since DNA metabarcoding of ice and firn samples was performed selecting methodological approaches to maximise DNA yield of these very diluted samples, controlling the DNA contamination was a strict requirement for the experiments. Negative controls were included in each step of the experimental procedure (concentration of samples, DNA extraction and DNA amplification). All negative controls were subjected to PCR and the PCR reaction was quantified by QIAxell, Qiagen.

Handling of samples was performed in distinct, separated and dedicated laboratories with specific requirements linked to the experimental step. Operators were dressed with disposable surgical gowns, gloves, masks and hand covers during the first phases of sample handling, concentration and DNA extraction, and lab surfaces and equipment were sterilized prior utilization. During the DNA extraction process, samples were aliquoted in a 96-plate, using a consistent plate layout, reducing the well-to-well contamination by using no-template blank wells and placing samples in alternated individual wells, both in rows and columns.

One negative control resulted positive at the DNA amplification step, and was sequenced. The contaminated control belonged to the concentration step of one of the samples, which was left inadvertently overnight in the switched-off concentrator. The control turned out to be contaminated by allochthonous plant DNA. Contaminating DNA was assigned to the unclassified taxa after bioinformatic classification against the local reference database. Search of unclassified contaminating sequences on public databases evidenced the presence of exogenous taxa like *Citrus sinensis* (389 reads), and *Phaseolus vulgari*s (77 reads), *Morus indica* (20 reads) and *Allium cepa* (17 reads). The major contaminant is likely accountable to a fragrance of a perfume present in the ambient air. The contaminating sequences did not affect the classification of the ice samples, based on the strict reference database that was used.
